# MicroRNA-588 regulates the invasive, migratory and vasculogenic mimicry-forming abilities of hypoxic glioma cells by targeting ROBO1

**DOI:** 10.1007/s11033-022-08063-z

**Published:** 2022-12-02

**Authors:** Rui Yu, Rongrong Zhao, Xiaopeng Sun, Zongpu Zhang, Shaobo Wang, Xiao Gao, Zhongzheng Sun, Hao Xue, Gang Li

**Affiliations:** 1 Department of Neurosurgery, Qilu Hospital, Cheeloo College of Medicine and Institute of Brain and Brain-Inspired Science, Shandong University, 107 Wenhua Xi Road, Jinan, 250012 Shandong China; 2 Shandong Key Laboratory of Brain Function Remodeling, Qilu Hospital, Jinan, 250012 Shandong China; 3 The Second Hospital, Cheeloo College of Medicine, Shandong University, Jinan, 250033 Shandong China; 4 Department of Neurosurgery, Qilu Hospital of Shandong University Dezhou Hospital, Dezhou, 253000 Shandong China; 5 Department of Vascular Surgery, Shandong Provincial Hospital Affiliated to Shandong First Medical University, 324 Jingwu and Weiqi Street, Jinan, 250021 Shandong China

**Keywords:** MicroRNA, Glioma, Motility, Hypoxia, Vasculogenic mimicry, Prognosis

## Abstract

**Background:**

The microenvironment of hypoxia is an important factor contributing to the development of glioblastoma (GBM). MicroRNA-588 and its potential target Roundabout-directed receptor 1 (ROBO1) have been reported to promote tumor invasion and proliferation in diseases such as gastric, pancreatic and hepatocellular carcinoma, while their function in GBM and response to hypoxic states remain elusive.

**Methods:**

A microarray was leveraged to identify differentially expressed microRNAs in U251 glioma cells cultured under normoxic and hypoxic conditions. The expression of miR-588 was assessed using quantitative real-time PCR (qRT‒PCR). Gain- and loss-of-function studies were used to evaluate the role of miR-588 under hypoxic and normoxic conditions. Cell invasion, migration, proliferation, and vasculogenic mimicry (VM) formation experiments were performed. The relationship between miR-588 and ROBO1 was confirmed using western blot and luciferase reporter assays. Intracranial xenograft tumor mouse models were used to study the function of miR-588 in vivo.

**Results:**

The expression of miR-588 was significantly upregulated in hypoxic glioma cells relative to normoxic glioma cells. miR-588 inhibited the invasive, migratory and VM-forming abilities of glioma cells in vitro and in vivo. Mechanistically, roundabout guidance receptor 1 (ROBO1) is a direct, functionally relevant target of miR-588 in glioma. ROBO1 knockdown suppressed the expression of matrix metallopeptidase 2 (MMP2) and matrix metallopeptidase 9 (MMP9), thereby inhibiting the invasive, migratory and VM-forming abilities of glioma.

**Conclusions:**

MiR-588 regulated the behaviors of hypoxic glioma cells by targeting ROBO1. miR-588 can be used as a prognostic marker for glioma and has potential implications in glioma gene therapy.

**Supplementary Information:**

The online version contains supplementary material available at 10.1007/s11033-022-08063-z.

## Introduction

Glioma is the most common tumor in the brain, accounting for 40–50% of all intracranial tumors [1]. The biological features of glioma mainly consist of a high fatality rate, high recurrence rate, strong invasive ability, strong angiogenic ability, and extensive hypoxic state [2].

Solid tumors, such as breast cancer, liver cancer and glioma, grow rapidly and correspondingly have an inadequate blood supply. A hypoxic microenvironment is prevalent in these tumors [3]. Tumor hypoxia is directly related to shorter survival times of patients [4]. To date, numerous studies have confirmed that hypoxia promotes tumor progression, including proliferation, invasion, migration and angiogenesis. The application of antiangiogenic drugs to treat glioma has attracted increasing attention [5]. However, according to recent studies, the clinical application of anti-vascular therapies is unsatisfactory due to their short-lived curative effect and poor efficacy [6].

Other blood supplies exist in tumor tissues in addition to blood vessels composed of endothelial cells. VM refers to channels that are functionally similar to normal blood vessels and are formed by tumor cells under specific conditions by mimicking vascular endothelial cells [7, 8]. These channels are connected to the tumor microcirculation, forming a vascular network to provide nutritional support to the tumors. Moreover, the amount of VM is strongly correlated with an advanced tumor grade as well as a poor prognosis. A hypoxic microenvironment is closely related to VM [9].

Although multiple mechanisms have been proposed to explain hypoxia-induced tumor cell invasion and migration, many researchers are currently investigating the roles of microRNAs (miRs) in the tumor hypoxia effect. MiRs are a series of endogenous, single-stranded noncoding RNAs with a length of 20–22 nucleotides. MiRs negatively regulate gene expression at the posttranscriptional level [10]. Previous studies focusing on miRs have revealed that they have key roles in the progression of tumors, such as proliferation, apoptosis, motility and angiogenesis [11].

Additionally, miR-588 has been reported to play a tumor suppressive role in lung cancer, breast cancer and other types of cancers [12–14]. In prostate cancer, miR-588 promotes tumor cell proliferation and functions as an oncogene [15]. However, its effects on the development and progression of glioma remain unclear. Our study identified miR-588 as a novel tumor suppressor and a valuable prognostic marker in glioma. Based on our in vitro experiments, miR-588 is capable of inhibiting invasion, migration and VM in glioma. Animal experiments revealed that miR-588 reduces the invasive and migratory capabilities of glioma cells by targeting hypoxia-induced roundabout guidance receptor 1 (ROBO1) expression. Thus, miR-588 exerts a tumor-suppressing effect on glioma. Our results emphasize the significance of miRs in tumor progression and identify the molecular mechanisms of action of miRs in tumor progression.

## Materials and methods

### Cell lines and tissue samples

The human glioma cell lines U251, U87MG, A172, human astrocyte cell (NHA) and human umbilical vein endothelial cell line (HUVECs) were acquired from the Chinese Academy of Sciences Cell Bank. The human glioma sections used in this study were verified in accordance with the World Health Organization tumor classification. Our study was approved by the Institutional Review Board of Shandong University and hospital ethical committee. This study was conducted on the basis of written informed consent obtained from all patients.

### Cells culture

Glioma cells and NHA were cultured using DMEM medium (Gibco, USA) containing 10% fetal bovine serum (FBS). HUVECs were maintained in Endothelial Cell Medium (ECM, Gibco, USA) containing 5% FBS and 1% Endothelial Cell Growth Supplement (ECGs, Gibco, USA). All cell lines were incubated at 37 °C, 5% CO_2_ and 21% O_2_ for normoxic research. For research under hypoxic conditions, cell culture was performed in modular culture chambers flushed with a gas mixture containing 1% O_2_, 5% CO_2_.

### Transfection

A mature miR-588 mimic and inhibitor were conceived and synthesized by RiboBio (Guangzhou, China). Transfection was performed with the assistance of lipofectamine 3000 according to the manufacturer’s protocol and scrambled oligos were used as negative controls. qRT-PCR was applied to detect the efficiency of transfection and subsequent assays were performed after 48 h of incubation of glioma cells.

### CCK-8 assay

We seeded U251, A172 and U87MG cells in 96-well plates at a density of 2 × 10^3^ cells per well. Cell propagation was assessed at 24, 48, 72, and 96 h post-transfection using Cell Counting Kit-8 (RiboBio, Guangzhou, China) following manufacturer’s protocol. The optical density values were recorded and analyzed at 450 nm.

### Wound-healing assay

We seeded about 1 × 10^5^ cells in 6-well plates overnight for cells adhesion. Then we performed cell transfection and when cells confluency reached 90 percent, a sterile pipette tip was used to scratched the cell monolayer. To reduce the impact of cell proliferation on the final results, we performed cell culture using serum-free medium after incisional wound formation. Representative images were obtained at 0 h and 36 h along the scrape lines and the relative scratch widths were recorded for subsequent analysis.

### Cell migration and invasion assays

Transwell chambers (Corning, 388767) were leveraged to conduct cell migration and invasion assays. In brief, about 5 × 10^4^ transfected cells were seeded in the upper chamber of FBS-free medium. Chambers with or without Matrigel were used for invasion and migration assays, respectively. The lower chamber was added with medium containing 20% FBS. The cells were fixed, stained and captured after 20 h (A172) or 6 h (U87MG) of migration or invasion. The numbers of migrating or invading cells were recorded randomly in five views using a microscope.

### VM formation assay

Matrigel Basement Membrane Matrix (0.1 ml/well, BD Bioscience, 354234) was used for coating the 24-well culture plate. The glioma cells were seeded on Matrigel at a density of 2 × 10^5^ cells/ml and cultured with Serum-free medium for 6 h. The representative images were collected using an inverted microscope.

### Human miRCURY™ LNA array analysis

U87MG cells were cultured under hypoxic or normoxic conditions and total RNA was extracted for microarray analysis using the TRIzol assay. The NanoDrop 1000 was used to evaluate the RNA quantities and miRCURY™ Hy3™/ Hy5™ Power Labeling Kit and hybridized to a miRCURY™ LNA Array (v.18.0) were used to label the RNA samples. The Axon GenePix 4000B microarray scanner was applied for the array scanning and the images were imported into GenePix Pro 6.0 software (Axon) for grid alignment and data extraction. Finally, the significant differentially expressed miRNAs among different groups were explored and validated.

### Luciferase reporter assay

TargetScan 5.2, miRDB and miRbase were leveraged to predict the potential targets of miR-588. The Reporter constructs containing pGL3-ROBO1 and pGL3-mutROBO1 were designed and constructed by Bio-Asia (Jinan, China). Cells were transfected as described previously, and 48 h later luciferase assays were performed with a luciferase assay kit according to the manufacturer's protocol.

### Real-time quantitative PCR

The TRIzol Reagent was leveraged for RNA extraction in accordance with manufacturer’s instruction. Then the extracted RNA was used to perform reverse transcription using a ReverTra Ace qPCR RT Kit. SYBR Premix Ex TaqTM Kit and the primers shown in Figure S1 were applied for real-time PCR. Finally, the absolute expression of RNA was analyzed and calculated by a Roche LightCycler® 2.0 system.

### Western blotting

RIPA buffer containing 1% phenylmethylsulfonyl fluoride was used for protein extraction and then loaded onto SDS–polyacrylamide gel for protein separation. The blots were incubated with primary antibodies against ROBO1 (Abcam, ab256791), GAPDH (Proteintech, 6004-1-lg), MMP-2 (CST, 40994) and MMP-9 (CST, 13667). Then the blots were incubated with secondary antibody at room temperature for 1 h and ECL detection system was used for protein visualization.

### Gelatin zymography experiment

The gelatin zymography experiment is widely used to detect the enzymatic activity of MMP-2 and MMP-9. Briefly, proteins obtained from different groups of cells were separated using SDS-PAGE on an 10% gel containing 1 mg/ml gelatin. The gels were then washed with 2.5% Triton X-100 and the MMPs were activated and enzymatically cleaved of their substrate when incubated in substrate buffer (50 mM Tris buffer containing 5 mM CaCl2). The gelatinolytic activity was visualized using Bio-RAD ChemiDoc XRS + system.

### Immunohistochemistry staining

4% formaldehyde was used for section fixation, and 1 mM EDTA (pH 8.0) was applied for antigen repair. The sections were incubated with goat serum for 2 h at room temperature, followed by overnight incubation with primary antibody (Abcam, ROBO-1, ab256791) at 4 °C. The sections were then incubated with the secondary antibody and then reacted with diaminobenzidine and counterstained with Mayer hematoxylin.

### In vivo experiments

We performed in vivo experiments on mice based on the approval of Institutional Animal Care and Use Committee of Qilu Hospital of Shandong University. Four-week-old male BALB/c nude mice were purchased from SLAC Laboratory Animal Center (Shanghai, China), and mice in similar conditions were randomly divided into different groups in preparation for the establishment of intracranial glioma xenograft models. Mice were anesthetized with 1% pentobarbital sodium (85.7 mg/Kg) and placed in a stereotactic frame. We made an incision in the parietal scalp of the mice and then drilled a small burr hole 2.5 mm lateral to bregma. U87MG luciferase cells (5 × 10^5^) transfected with lenti-control, lenti-miR-588 or lenti-sh-miR-588 virus were extracted and injected into the skull. The above procedures were performed under aseptic surgical conditions. Tumor growth was monitored by intraperitoneal injection of 150 mg/kg of fluorescein followed by bioluminescence with an IVIS Lumina Series III (PerkinElmer). We euthanized mice when they developed severe neurological symptoms or turned moribund. We strictly implemented the "3R principles of animal experimentation" in our animal experiments.

### Statistical analysis

All experiments were conducted three times. SPSS 17.0 and GraphPad Prism software were leveraged for statistical analyses and experimental graphs. Mann–Whitney test was used for comparisons of two groups, while non-parametric Kruskal–Wallis test was used for multiple comparisons. Kaplan–Meier curves and log-rank test were used to visualized and assessed survival information. One-way ANOVA and Spearman’s correlation test were leveraged to analyze the significant differences, and a *p < 0.05, **p < 0.01 were regarded as statistically significant.

## Results

### Experimental analysis of the differential expression of miRs in glioma cell lines cultured under hypoxic and normoxic conditions

**Fig. 1:**
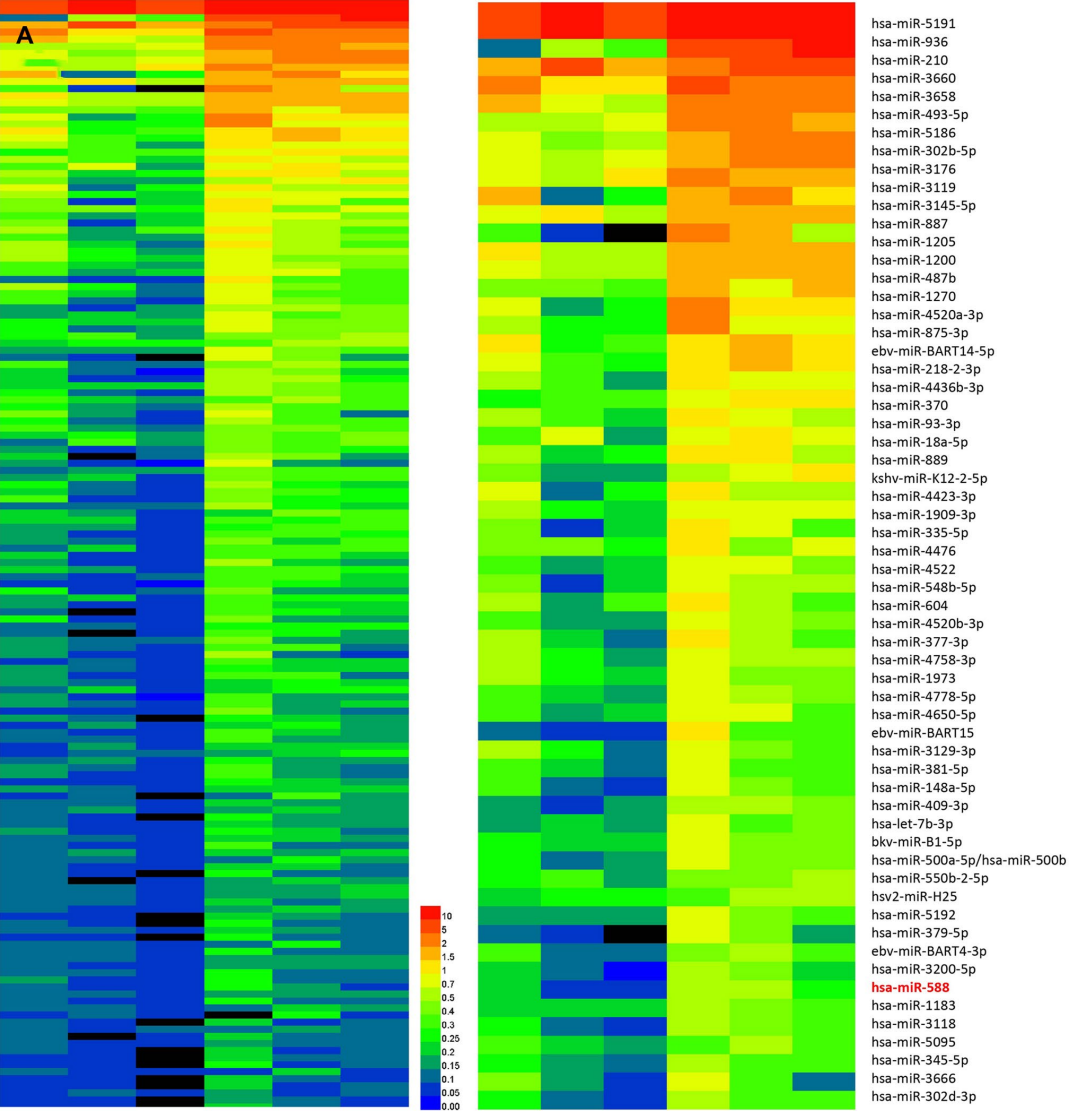

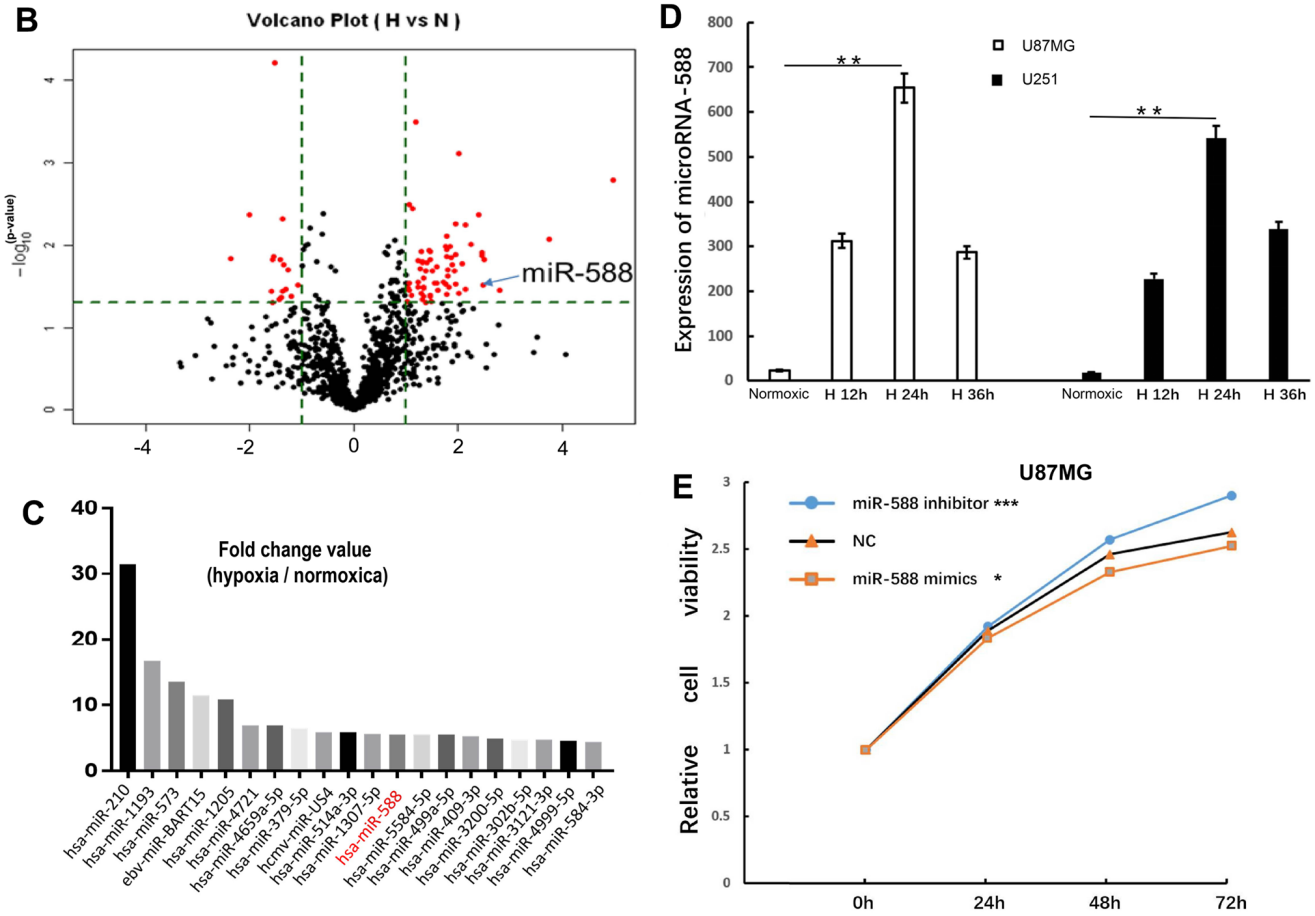
miRNA array analysis of differentially expressed miRNAs in hypoxic glioma cell lines and miR-588 expression levels in human glioma tumors and cell lines. **A** miRCURY™ LNA expression array revealed 84 significantly differentially expressed miRNAs (partial data in figure) between normoxic and hypoxic U251 cells. The hypoxic miRNA marker miR-210 and the target miRNA miR-588 are indicated. **B** Volcano plots were constructed to screen the differentially expressed miRNAs between the two conditions according to the fold changes and *p*-values. The vertical lines correspond to 2.0-fold changes (either up or down), and the horizontal line represents a *p*-value of 0.05. The red dots in the plot represent the significantly differentially expressed genes, including miR-588. **C** miR-588 was among the top ten modulated genes in hypoxic glioma cells, and it was up-regulated (according to the fold change). **D** The expression levels of miR-588 in hypoxic U251 and U87MG cells (hypoxia treatment for 0, 12, 24, and 48 h) were assessed by quantitative real-time PCR. **p < 0.01, *p < 0.05 by Kruskal–Wallis one-way ANOVA. **E** Cell viability assay of U251 cells transfected with NC, the miR-588 mimics and inhibitor

Hypoxia is an important contributor to a shorter survival of glioma patients. High-throughput whole-genome miR screening was conducted in U251 glioma cells cultured under hypoxic and normoxic conditions using miR microarray chips to investigate hypoxia-induced differences in miR expression. The expression of numerous miRs was significantly upregulated under hypoxic conditions (Fig. 1A). Among the significantly differentially expressed miRs, miR-210, which showed the greatest upregulation, has been previously confirmed to be a hypoxia marker. Additionally, we previously cultured the U87MG and A172 cells under hypoxia conditions and detected the microRNAs expression. The results demonstrated that miR-588 and miR-210 favorably reflected the results of microarray chips in U251, whereas other microRNAs were not particularly satisfactory (Figure S1A). In addition, we used a volcano plot to analyze the microarray data and confirmed that miR-588 was significantly upregulated under hypoxic conditions (Fig. 1B).

We then examined the transfection efficiencies of NC, miR-588 mimics and a miR-588 inhibitor using RT‒qPCR. The expression of miR-588 was not affected in cells transfected with NC. In contrast, transfection of the mimics significantly increased the expression of miR-588, whereas transfection of the inhibitor markedly inhibited the expression of miR-588. Subsequently, RT‒qPCR was employed again to examine the expression of miR-588 in U251 and U87MG cells cultured under normoxic conditions and different durations of hypoxia. The expression of miR-588 was significantly upregulated under hypoxic conditions compared to normoxic conditions (Fig. 1D). Moreover, miR-588 expression peaked in glioma cells cultured under hypoxic conditions for approximately 24 h. We further detected the expression of miR-588 expression in glioma cells and normal human astrocytes (NHA). The results showed that miR-588 was highly expressed in NHA cell line compared to glioma cell lines (Figure S1B).

NC, miR-588 mimics and the miR-588 inhibitor were used in the present study. NC was the negative control RNA. Transfection of mimics leads to overexpression of the relevant RNAs. MiR inhibitors are synthetic single-stranded RNA molecules that inhibit the functions of the corresponding miRs through irreversible binding [16, 17]. In the present study, an miR inhibitor was used to block miR-588 function. U87MG cells were stably transfected with lentiviral vectors expressing NC, miR-588 mimics and the miR-588 inhibitor. Cell Counting Kit-8 (CCK8) assays were applied to observe the effects of these molecules on cell viability. No significant differences in viability were observed among glioma cells transfected with NC, miR-588 mimics and miR-588 inhibitors. Thus, stable transfection with 80 nM mimics and inhibitors significantly regulated endogenous miR-588 expression without affecting cell viability.

### The expression of miR-588 is implicated in the invasive, migratory and VM-forming abilities of hypoxic glioma cells

**Fig. 2:**
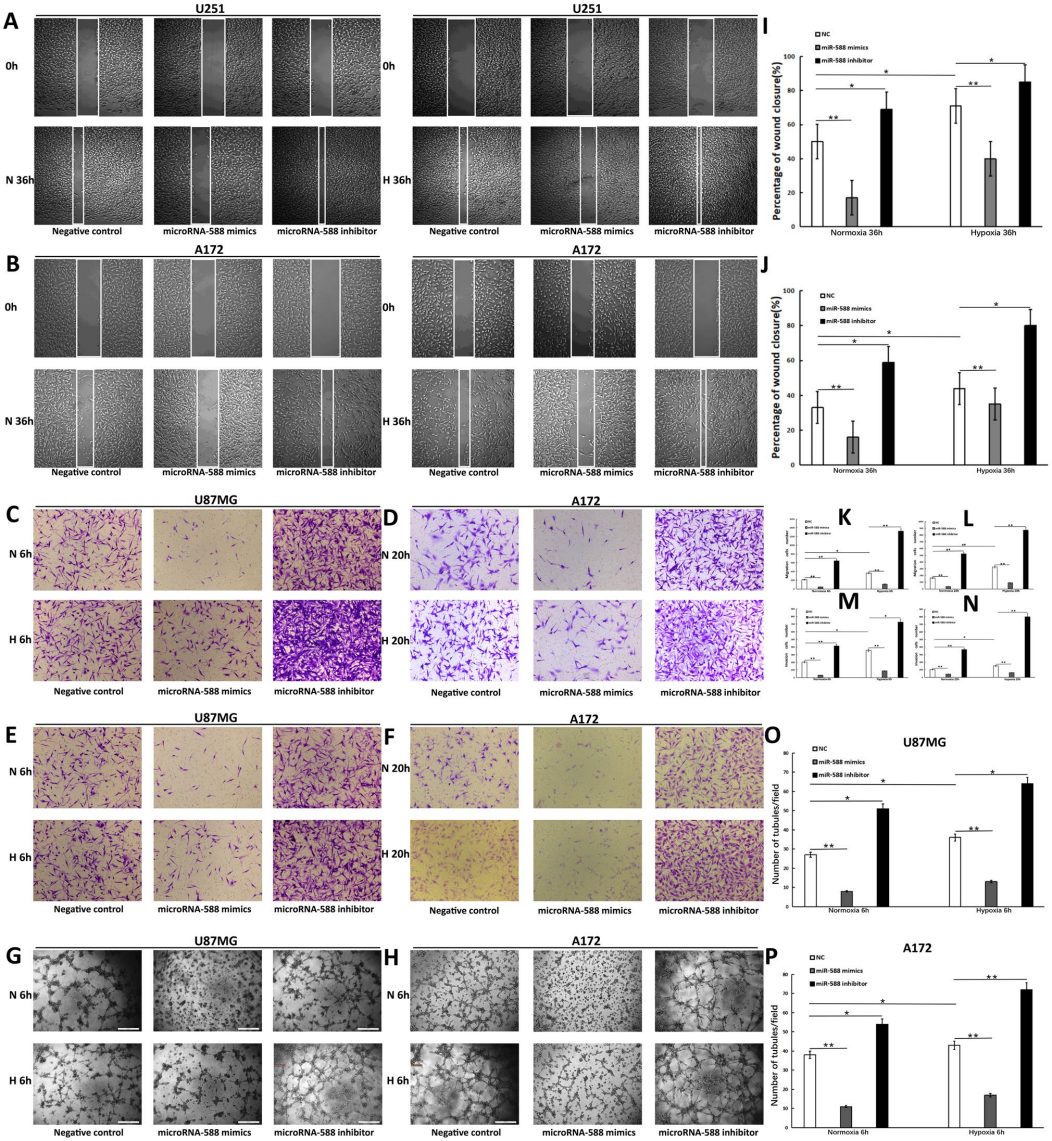
The overexpression of miR-588 inhibited and miR-588 knockdown enhanced the invasive, migratory and VM-forming abilities of hypoxic glioma cells. **A** and **I** Wound-healing assay of U251 cells transfected with miR-588 mimics and inhibitor under normoia and hypoxa. **B** and **J** Wound-healing assay of A172 cells transfected with miR-588 mimics and inhibitor under normoia and hypoxa. **C** and **K** The regulating effect of the miR-588 mimics and inhibitor on U87MG cell migration under normoxia and hypoxia was examined by Transwell migration assays. **D** and **L** The regulating effect of the miR-588 mimics and inhibitor on A172 cell migration under normoxia and hypoxia was examined by Transwell migration assays. **E** and **M** The regulating effect of the miR-588 mimics and inhibitor on U87MG cell invasion under normoxia and hypoxia was examined by Matrigel invasion assays. **F** and **N** The regulating effect of the miR-588 mimics and inhibitor on A172 cell invasion under normoxia and hypoxia was examined by Matrigel invasion assays. **G** and **O** The regulating effect of the miR-588 mimics and inhibitor on U87MG cell VM-forming ability under normoxia and hypoxia was examined by VM formation assays. **H** and **P** The regulating effect of the miR-588 mimics and inhibitor on A172 cell VM-forming ability under normoxia and hypoxia was examined by VM formation assays. At 48 h after transfection, a cell suspension was added to a Matrigel-coated well of 96-well tissue culture plate, then take pictures every 2 h. **p < 0.01, *p < 0.05 by Kruskal–Wallis one-way ANOVA

The glioma cell lines U87MG, U251 and A172 were stably transfected with NC, miR-588 mimics and miR-588 inhibitors in the current study to examine whether the upregulation or downregulation of miR-588 regulated the hypoxia-induced migration, invasion and VM formation of glioma cells. Scratch assays were performed using the A172 and U251 cell lines to examine the effects of miR-588 on glioma cell migration (Fig. 2A, B).

Several interesting phenomena were observed in the present study. First, cell migration was significantly increased under hypoxic culture conditions. This result was consistent with our expectations. Second, miR-588 significantly suppressed the migration of glioma cells and substantially antagonized hypoxia-induced migration. The miR-588 knockdown glioma cells displayed a significant increase in migration, and hypoxic culture further promoted cell migration. Both the level of miR-588 and the oxygen level in the incubator regulated the migration of glioma cells. Moreover, miR-588 and oxygen exerted mutual regulatory effects.

Transwell assays were performed using the U87MG and A172 cell lines to evaluate further the effect of miR-588 on the migration and invasion of glioma cells (Fig. 2C–F). U87MG cells displayed significantly increased migration through the polycarbonate membrane compared to A172 cells. Based on the pilot experiments, U87MG cells were fixed and stained after 6 h of cultivation in Transwell chambers, whereas A172 cells were fixed and stained after 20 h of cultivation. Nevertheless, similar results were obtained using the two cell lines. In comparison with the control group, miR-588 significantly suppressed the ability of glioma cells to migrate through the membrane, whereas the miR-588 inhibitor markedly enhanced the migratory capability of glioma cells. In addition, hypoxia also significantly promoted the transmembrane migration of glioma cells. Subsequently, we examined the invasion of glioma cells using chambers coated with Matrigel and obtained results similar to those of the migration assays.

Thus, miR-588 significantly suppressed the invasion and migration of glioma cells and simultaneously antagonized the promotion of invasion and migration by hypoxia. Knockdown of miR-588 significantly enhanced the invasion and migration of glioma cells.

The U87MG and A172 cell lines were cultured in Matrigel to examine the development of VM [18, 19]. During the experiment, the glioma cells first aggregated and extended protrusions, which were then connected to form tubular structures (i.e., VM). The tubular structures collapsed after a certain period. Based on these results, hypoxia accelerated the rate of VM formation and increased the number of tubular structures formed. In comparison with the control group, overexpression of miR-588 significantly inhibited the rate of VM formation and decreased the number of tubular structures. In addition, overexpression of miR-588 antagonized hypoxia-induced VM formation. In contrast, the miR-588 knockdown cells displayed a remarkable increase in VM formation (Fig. 2G, H). VM formation was consistent with the invasion and migration of the cells. Moreover, miR-588 significantly inhibited the VM-forming ability of glioma cells.

We further performed VM-forming experiment using HUVEC cells. As the results demonstrated, transfection of miR-588 mimics promoted the VM-forming ability of cells, whereas transfection of miR-588 inhibitors inhibited the VM-forming ability (Figure S1D).

### ROBO1 is a key downstream gene in the mechanism by which miR-588 inhibits the invasion, migration and VM-forming abilities of glioma cells

**Fig. 3:**
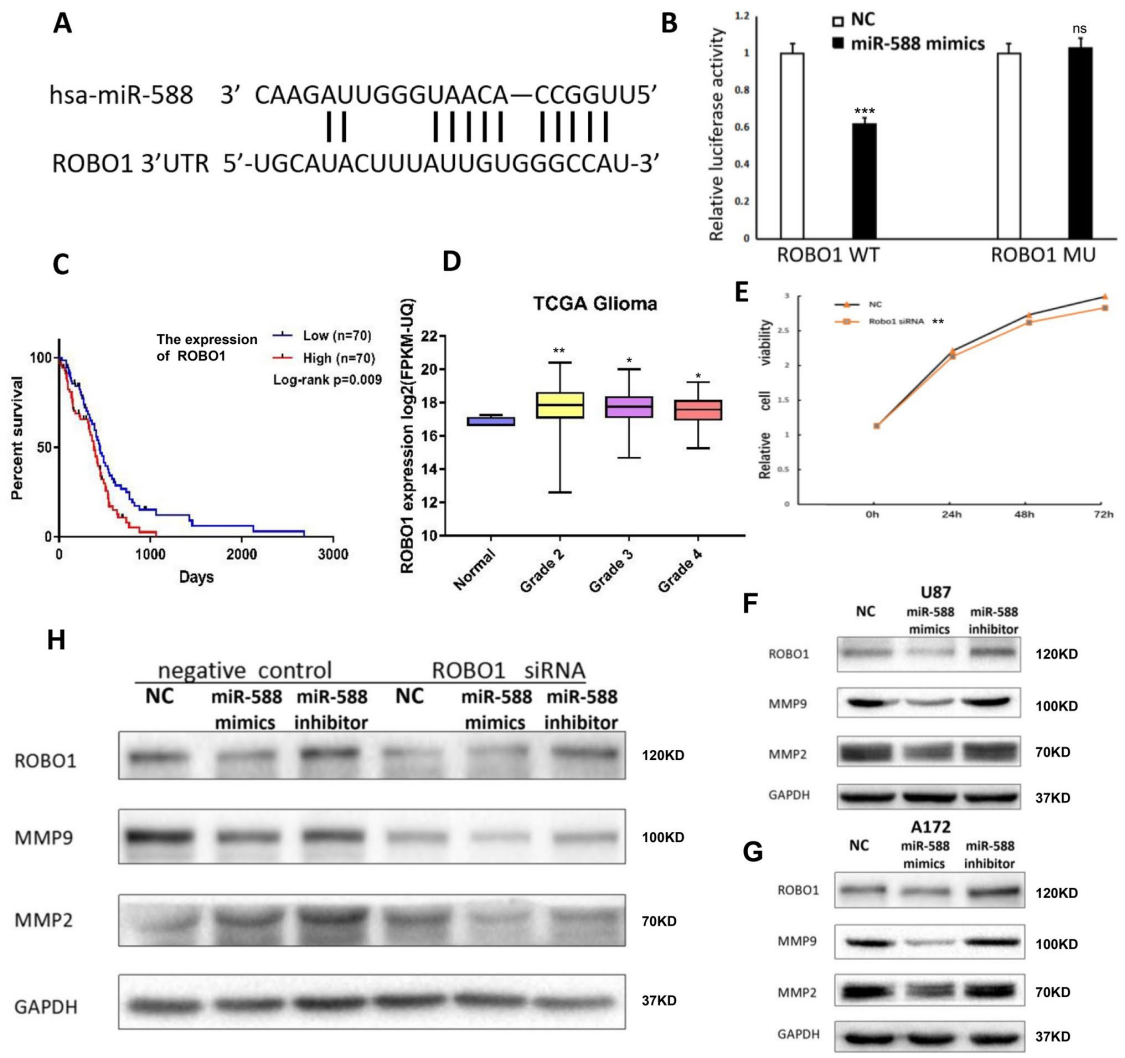
ROBO1 is a key downstream gene of miR-588. **A** Sequence of the miR-588 binding site in ROBO1, the miR-588 binding site in the ROBO1 3′-UTR was predicted using Targetscan. **B** Results of 3′-UTR luciferase assay.. A 3′-UTR vector and miR-588 or miRNA negative control were co-transfected into U87MG cells. The luciferase activity levels were compared with those of the miRNA negative control-transfected cells, which were normalized to 1. **C** Patients with glioma expressing a high level of ROBO1 experienced significantly shorter survival. **D** ROBO1 expression was significantly increased in gliomas compared with normal brain tissues. **E** Cell viability assay of U87MG cells transfected with NC and ROBO1 siRNA. **F** The ROBO1 protein levels in U87MG cell transfected with miR-588. GAPDH was used as a loading control. **G** The ROBO1 protein levels in A172 cell transfected with miR-588. **H** The ROBO1 protein levels in U87MG cells stably transfected with NC, mimics and inhibitor and transfected with ROBO1 siRNAs. GAPDH was used as a loading control

The key downstream target genes of miR-588 must be identified to clarify the mechanisms by which miR-588 regulates the invasion, migration and VM-forming abilities of glioma cells. We searched for genes that were downstream of miR-588 and associated with the invasion and migration of glioma cells using the TargetScan, miRDB and miRBase databases. After an integrated analysis of three datasets, we identified 3 candidate genes, which were ROBO1, FOXA1 and IGDCC4 (Figure S2A). We further detected their expression after knockdown and overexpression of miR-588 using using RT‒qPCR (Figure S2B). The results indicated that ROBO1 was the most likely target of miR-588. Additionally, the correlation analysis based on CGGA dataset showed that the expression of miR-588 was negatively correlated with ROBO1 (Figure S2C). The three prime untranslated region (3ʹ-UTR) of the ROBO1 gene contains target sites that directly bind miR-588 (Fig. 3A). ROBO1 is a conserved transmembrane receptor protein that is primarily expressed in the nervous system. An analysis of The Cancer Genome Atlas (TCGA) database demonstrated that ROBO1 expression was significantly elevated in glioma tissue in comparison with normal brain tissue, and patients with glioma expressing a high level of ROBO1 experienced significantly shorter survival (Fig. 3C, D). Based on these results, ROBO1 promotes the development of gliomas. After transfection with miR-588 mimics and inhibitors for 48 h, proteins were extracted from the glioma cells, and western blot analysis was performed to examine the inhibitory effect of miR-588 at the protein level. Overexpression of miR-588 reduced ROBO1 levels, whereas knockdown of miR-588 increased the levels of the ROBO1 protein (Fig. 3H). Therefore, miR-588 regulated ROBO1 expression and was negatively correlated with ROBO1 expression. A luciferase reporter assay was performed to validate the association between miR-588 and ROBO1. In comparison with the control group, luciferase activity was reduced to approximately 61.3% in cells transfected with miR-588, suggesting that the expression of ROBO1 was directly modulated by miR-588 in glioma cells (Fig. 3B).

Western blots also showed that miR-588 overexpression downregulated the expression of matrix metalloproteinase-2 (MMP2) as well as matrix metalloproteinase-9 (MMP9) in U87MG and A172 cell lines (Fig. 3F, G). MMP2 is positively correlated with the invasion and migration of glioma cells, while MMP9 is implicated in cell invasion, migration and VM formation. Additionally, gelatin zymography experiments were performed to detect the activity of MMP-2 and MMP-9. Transfection of miR-588 mimics significantly inhibited the activity of MMP-2 and MMP-9, while the opposite result was observed for transfection of miR-588 inhibitors (Figure S1B). Therefore, the above results partially illustrated the mechanisms by which miR-588 inhibited the invasion, migration and VM-forming abilities of glioma cells. No change in the viability of cells stably transfected with the lentiviruses was detected using the CCK8 assay. The stably transfected U87MG cells were then transfected with a small interfering RNA (siRNA) targeting ROBO1. Following siRNA transfection, cellular proteins were extracted. ROBO1 expression was significantly suppressed in cells transfected with ROBO1 siRNA compared with the control group, demonstrating that the efficiency of siRNA-mediated knockdown of ROBO1 expression was satisfactory. In addition, western blot analysis revealed a simultaneous decrease in MMP2 and MMP9 levels after ROBO1 knockdown.

These results demonstrated that the reduction of ROBO1 expression inhibited the expression of MMP2 and MMP9, thereby affecting the invasive, migratory and VM-forming abilities of glioma cells. These results partially demonstrated the mechanisms by which miR-588 regulated the invasion, migration and VM formation of gliomas.

### The invasive, migratory and VM-forming abilities of glioma cells were decreased after ROBO1 knockdown

**Fig. 4:**
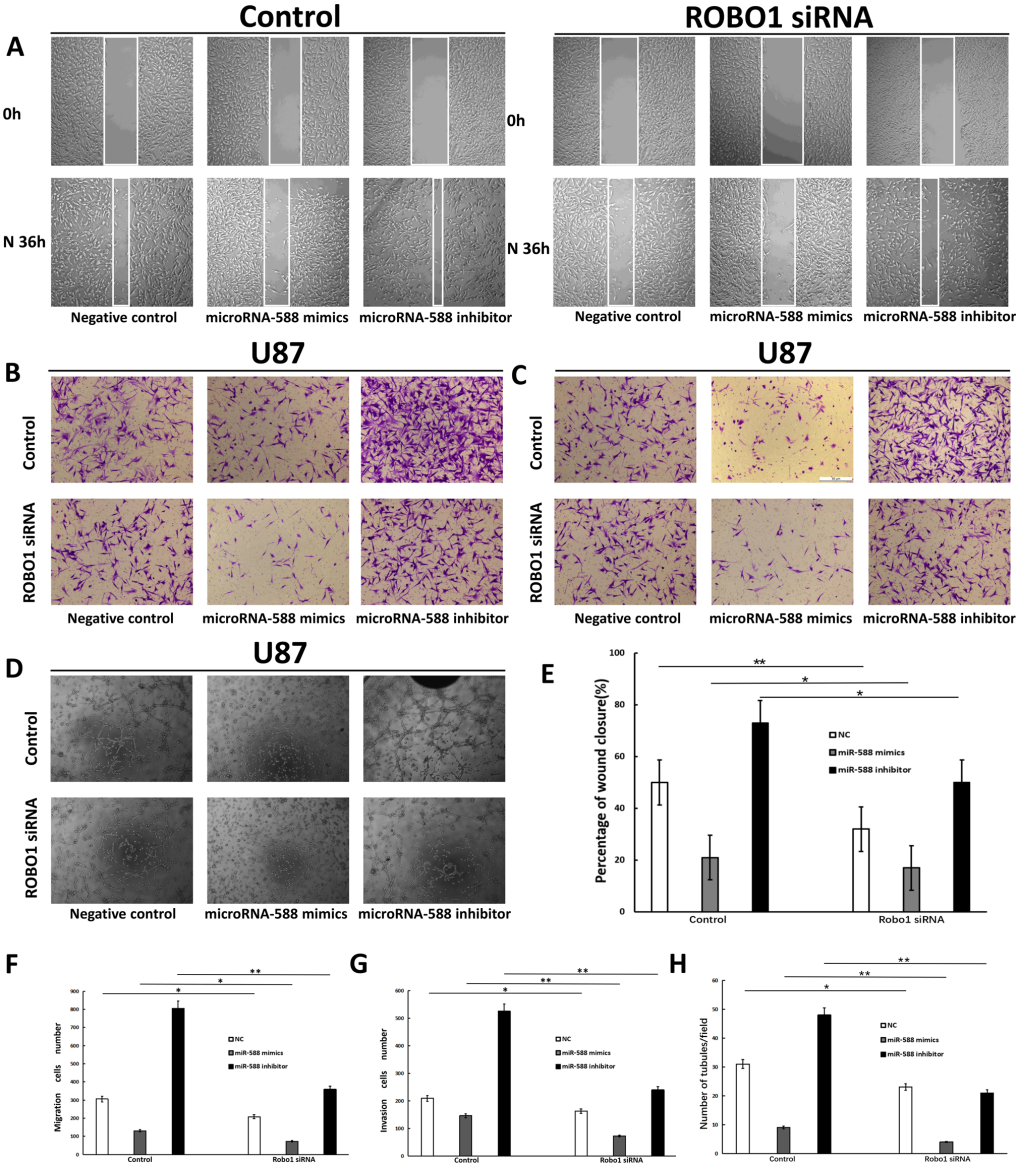
The invasive, migratory and VM-forming abilities of glioma cells were decreased after ROBO1 knockdown. **A** and **E** Wound-healing assays of miR-588 inhibitor- and mimics-transfected U87MG cells co-transfected with ROBO1 siRNA. **B** and **F** Effects of ROBO1 siRNA on miR-588 inhibitor- and mimics-transfected U87MG cells were examined by Transwell migration assays. **C** and **G** Effects of ROBO1 siRNA on miR-588 inhibitor- and mimics-transfected U87MG cells were examined by Transwell invasion assays. **D** and **H** Effects of ROBO1 siRNA on miR-588 inhibitor- and mimics-transfected U87MG cells were examined by VM formation assays. **p < 0.01, *p < 0.05 by Kruskal–Wallis one-way ANOVA

U87MG glioma cells stably transfected with NC, miR-588 mimics or the miR-588 inhibitor were further transfected with ROBO1 siRNA to verify the effects of ROBO1 on glioma cell invasion, migration and VM. After transfection, the cells were subjected to scratch, Transwell and VM assays. Cells displaying normal ROBO1 expression served as the control group. In comparison with the control group, the migration of glioma cells in the scratch assay was significantly reduced after ROBO1 knockdown. Cells stably transfected with miR-588 mimics displayed the lowest migration ability after ROBO1 knockdown. Cells stably transfected with the miR-588 inhibitor in which ROBO1 expression was knocked down exhibited similar migration to NC-transfected cells, which was significantly suppressed in comparison with the control group with normal ROBO1 expression (Fig. 4A).

Similar findings were observed in the subsequent Transwell assay (Fig. 4B, C). Normally, knockdown of ROBO1 expression significantly suppresses the migration of glioma cells. In addition, the results from the invasion assay were in accordance with the findings of the migration assay. ROBO1 knockdown also inhibited the invasion of glioma cells. Based on these results, ROBO1 significantly increased the invasion and migration of glioma cells, while ROBO1 knockdown enhanced the ability of miR-588 to inhibit glioma cell invasion and migration. ROBO1 served as a downstream target of miR-588. Moreover, miR-588 exerted its cancer-suppressing effect by inhibiting ROBO1 expression.

A VM formation assay was conducted using glioma cells transfected with ROBO1 siRNA to clarify the effect of ROBO1 on the development of VM in glioma cells. Compared with the control group, ROBO1 knockdown cells exhibited a delay in VM formation. In addition, knockdown of ROBO1 expression markedly reduced the number of tubular structures formed (Fig. 4D). Thus, the VM-forming capacity of glioma cells was markedly reduced by ROBO1 knockdown.

We concluded that ROBO1 also plays a vital role in the formation of VM structures in glioma cells. The above results were in accordance with the findings of the invasion and migration assays.

### Overexpression of ROBO1 inhibited the function of miR-588 in vitro and in vivo

To further validate this conclusion that miR-588 exerts its function by targeting ROBO1, we overexpressed ROBO1 in glioma cells transfected with miR-588 mimics. As the results demonstrated, overexpression of miR-588 significantly promoted the invasiveness, migration and VM formation of U87MG glioma cells, while overexpression of ROBO1 could curtail the effect of miR-588 overexpression (Figure S2D–F). We further performed in vivo experiment using nude mice. Despite the initial tumor size being similar, xenografts bearing miR-588-U87MG cells exhibited tumor growth inhibition and longer survival times than control group, whereas overexpression of ROBO1 inhibited the effect of miR-588 in vivo (Figure S3C, D).

### ROBO1 expression in glioma tissues was positively correlated with VM formation

**Fig. 5:**
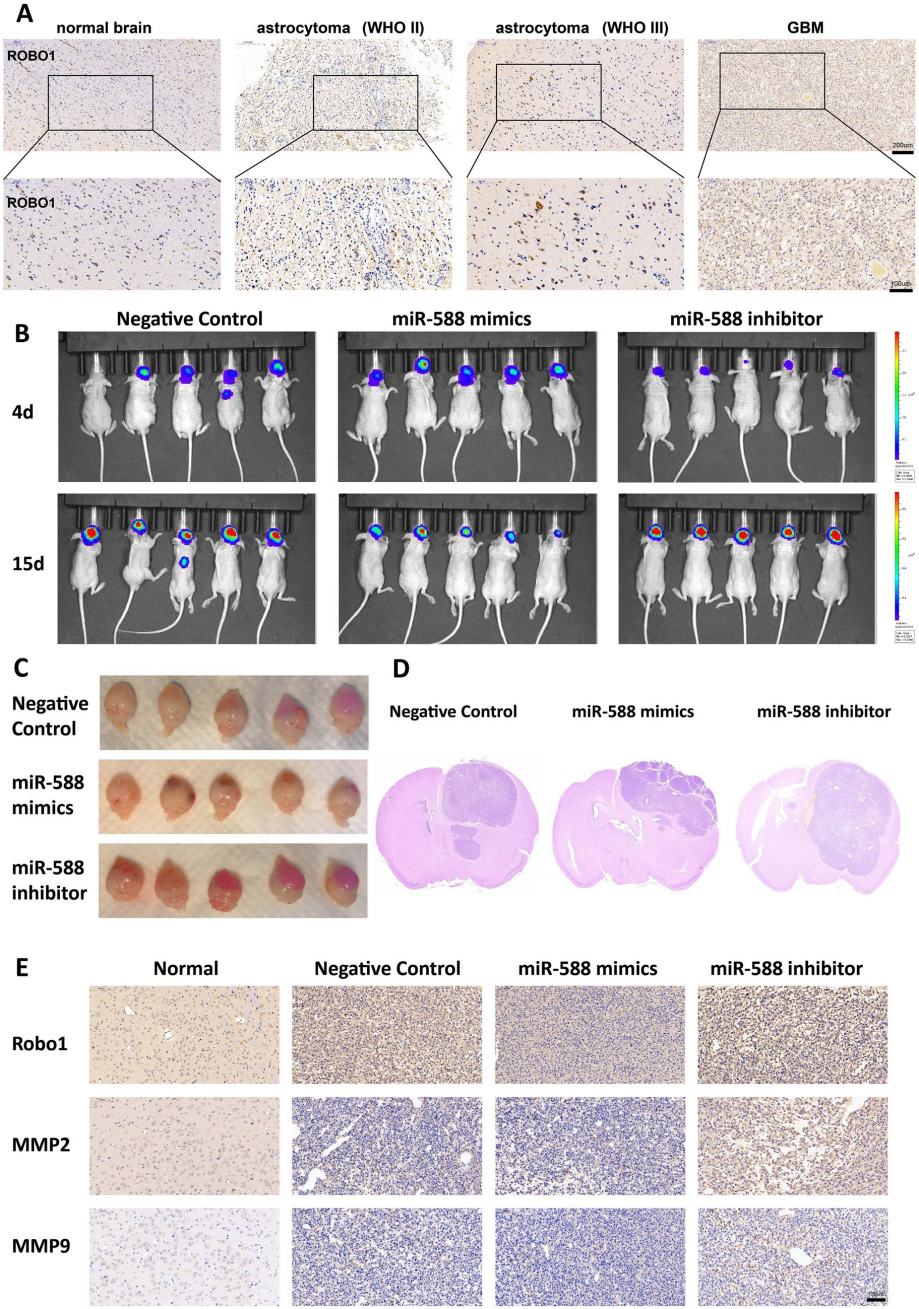
miR-588 antagonizes hypoxia-induced pro-invasive effects in an orthotopically xenografted glioma mouse model. **A** ROBO1 expression gradually increased in the glioma compared with normal brain. **B** Bioluminescence images of the sizes of the intracranial tumors in the model mice on day 4 and day 15. **C** Mice were sacrificed for brain removal 21 days after tumor implantation. **D** Hematoxylin and eosin (H&E) staining of orthotopically xenografted glioma. **E** Immunohistochemical staining showed ROBO1 increased in xenografted glioma with miR-588 inhibitor transfected, MMP2 and MMP9 rised up also

Normal brain tissue specimens and specimens of various grades of glioma tissues (WHO grades II-IV) were collected. Subsequently, ROBO1 immunohistochemistry, CD31 immunohistochemistry and periodic acid–Schiff (PAS) staining were performed to examine the differences in ROBO1 expression and VM formation among the various grades of glioma tissues and to determine the potential correlation between ROBO1 expression and VM formation. ROBO1 expression gradually increased as the glioma grade increased (Fig. 5A). At the same time, VM formation increased. Therefore, a positive correlation was identified between ROBO1 expression and VM formation.

### miR-588 counteracts the hypoxia-induced pro-invasive effects in vivo

To analyze the impact of miR-588 on hypoxia-induced invasion in vivo, we transplanted lentiviral vector-transfected U87MG cells into the brains of nude mice. We detected and recorded the intracranial tumor size by bioluminescence imaging. The results demonstrated that miR-588 knockdown tumors were significantly more aggressive, while miR-588 overexpression strongly reduced the extent of growth of the gliomas (Fig. 5B, C and Figure S3A). Hematoxylin and eosin (HE) staining suggested an extremely aggressive status and a marked increase in tumor growth of the miR-588 knockdown xenografted glioma relative to the NC group (Fig. 5D). Overexpression of miR-588 leads to remarkable inhibition of glioma cell invasion and proliferation. IHC staining demonstrated a negative correlation between miR-588 and ROBO1 expression. These results revealed that ROBO1 was barely detectable in normal brains but was potently upregulated in xenograft tumors after miR-588 knockdown (Figs. 5E and S3B). Meanwhile, we discovered that MMP2 and MMP9 levels increased in the miR-588 knockdown tumors. Therefore, miR-588 deficiency significantly facilitated the overall invasive ability of glioma cells in this mouse model. The above results are compatible with the unfavorable prognosis of clinical survival in glioma patients exhibiting low miR-588 levels.

## Discussion

Glioma is the most challenging intracranial malignancy, as it is the most common and most difficult tumor to cure [20]. The explanations for the high degree of malignancy of glioma include the hypoxic microenvironment, highly invasive and migratory abilities, abundant blood vessels, VM formation and chemoradiotherapy resistance, which have all been the focus of research in recent years [11].

The culture of glioma cells under hypoxic conditions leads to specific biological responses, comprising the activation of signaling pathways responsible for regulating propagation, angiogenesis, metastasis and apoptosis. The hypoxic tumor environment is related to a poor prognosis, radiotherapy resistance and chemotherapy resistance [21, 22]. In surgically resected glioma tissues, living cells surround the central necrotic tissues and migrate outward, indicating that hypoxia might regulate this process [4, 23]. According to the results from in vivo and in vitro assays, tumor hypoxia enhances the invasion and migration of glioma cells. However, the specific mechanisms remain unclear.

A variety of miRs have recently been shown to participate in this process. In the field of glioma research, miR-based treatments have been explored for approximately 10 years and are expected to make a major breakthrough. In recent years, several miRs that are expressed at high levels in glioma (such as miR10b, miR21, miR210 and miR221/222) have been identified as markers of a poor prognosis [24–27]. In addition, a number of tumor suppressor miRs have been identified, including miR663 and miR128. In the present study, miR microarray technology was employed to compare miR expression between glioma cells cultured under normoxic and hypoxic conditions. A number of miRs whose expression was upregulated under hypoxic conditions were identified and considered related to the hypoxic microenvironment of glioma. These miRs were further screened using in vitro assays.

Notably, miR-588 inhibited the invasion and migration of glioma cells. In some previous studies, miR-588 functioned as a tumor suppressor in lung cancer, gastric cancer and breast cancer. The mechanisms by which miR-588 suppresses cancer growth include inhibition of invasion, migration, and angiogenesis and induction of apoptosis [12–14]. In prostate cancer, miR-588 promotes tumor cell proliferation and functions as an oncogene [15]. The effect of miR-588 in glioma has not been reported previously. Therefore, we decided to conduct an in-depth study of miR-588.

Glioma is a highly vascularized brain tumor. However, the current anti-vascular therapies display a short-lived curative effect and poor efficacy in clinical practice [28, 29]. With the proposition of the concept of VM in 1999, researchers recognized that other blood supplies exist in tumor tissues in addition to the blood vessels composed of endothelial cells [30, 31]. VM refers to channels that are functionally similar to normal blood vessels and are formed by tumor cells under specific conditions by mimicking vascular endothelial cells [32]. These channels are connected to the tumor microcirculation, forming a vascular network to provide nutritional support to the tumors.

VM has the following characteristics: (1) tubular structures are composed of tumor cells rather than endothelial cells and are connected to the tumor microcirculation system, (2) extracellular matrix (ECM) remodeling occurs, and (3) PAS staining and negative CD31 staining are observed [33]. VM occurs in a variety of progressive tumors, including glioma, and a higher degree of VM is strongly associated with the tumor grade and a poor prognosis of patients [18]. Patients with high levels of VM are more prone to tumor metastasis and have a lower survival rate. Therefore, a therapeutic strategy targeting VM may be used a promising antitumor vascular therapy.

VM formation occurs mainly through glioma stem cells and the transforming growth factor beta (TGFβ), vascular endothelial growth factor receptor 2 (VEGFR-2)/fetal liver kinase 1 (FLK-1), vascular endothelial cadherin (VE-cadherin), receptor tyrosine kinase (RTK)/phosphoinositide 3-kinase (PI3K)/AKT/mammalian target of rapamycin (mTOR), and MMP-laminin5γ2 chain pathways [33, 34]. Both epithelial-mesenchymal transition (EMT) and a hypoxic microenvironment act as key players in VM formation. In the present study, miR-588 was significantly upregulated under hypoxic conditions, and overexpression of miR-588 suppressed the invasion and migration of glioma cells. Based on these findings, miR-588 is related to VM formation in glioma. A VM formation assay was conducted in the present study using glioma cells, and the findings demonstrated that miR-588 inhibited VM formation by glioma cells. Thus, as a tumor suppressor, miR-588 represents a promising prognostic biomarker for progressive glioma. Therefore, miR-588 is worthy of further investigation.

Few reports have investigated the pathways involved in the mechanisms by which miR-588 suppresses tumor growth. In recent studies, we attempted to elucidate the implications of miR-588 in hypoxia. Based on the findings of our previous study, we hypothesized that miR-588 antagonizes the hypoxia-induced progression of glioma and inhibits the invasion, migration and VM-forming abilities of tumors. Preliminary experimental results showed that the motility and VM-forming ability were markedly reduced in cells transfected with miR-588 mimics. Knockdown of miR-588 yielded the opposite result. Moreover, the impacts of miR-588 were amplified under hypoxic conditions. The expression of miR-588 was upregulated in response to hypoxia and exerted a cancer-suppressing effect. Therefore, we postulate that miR-588 is associated with a protective compensatory mechanism and plays a key role in suppressing the hypoxia-induced invasion, migration and VM formation of glioma cells.

We screened for downstream target genes of miR-588 using three target gene prediction websites [35–37]. The motility-related gene ROBO1 was identified as a potential oncogene target, which was experimentally verified. ROBO1 is a conserved transmembrane receptor protein. It is mainly expressed in the nervous system and is implicated in the transmission of biological information [38].

The roles of ROBO1 in tumor development and progression remain unclear. ROBO1 has been reported to function as both an oncogene and a tumor suppressor [39]. An analysis of the TCGA database revealed a significant increase in ROBO1 expression in gliomas compared to normal brain tissues [40]. Moreover, the survival of patients with glioma who expressed ROBO1 at high levels was significantly shortened. The luciferase reporter assay demonstrated a direct target interaction between miR-588 and ROBO1. Western blot analyses indicated that miR-588 simultaneously decreased the expression of ROBO1, MMP2 and MMP9 (markers of invasion, migration and VM). Further cell-based experiments revealed a decrease in the invasion, migration and VM-forming abilities of glioma cells after ROBO1 knockdown. In addition, ROBO1 knockdown inhibited the ability of the miR-588 inhibitor to promote invasion and migration.

We propose the mechanisms of action of miR-588: In a hypoxic environment, miR-588 binds directly to the 3'-UTR to downregulate ROBO1 expression. MMP2 and MMP9 levels are also reduced. Consequently, the invasion, migration and VM-forming abilities of the cells are inhibited (Figure S3E). Notably, miR-588 is a hypoxia-induced tumor suppressor that is capable of antagonizing the invasion-promoting effect of hypoxia, consistent with our clinical observations. Other molecules regulating ROBO1 expression and target molecules of miR-588 may also play roles in this complex process, requiring further investigation.

## Conclusions

In summary, this study described a microRNA, miR-588, that targets ROBO1 to inhibit invasion, migration and VM formation of glioma. We propose that miR-588 represents a prognostic biomarker for glioma and is a promising therapeutic target for the treatment of glioma.

## Supplementary Information

Below is the link to the electronic supplementary material.

Supplementary file1 (DOCX 1793 kb)

## Data Availability

All the data obtained and/or analyzed during the current study were available from the corresponding authors on reasonable request.
