# Supplementary material for: MicroRNA-588 regulates the invasive, migratory and vasculogenic mimicry-forming abilities of hypoxic glioma cells by targeting ROBO1
Source: Mol Biol Rep. 2022 Dec 2;50(2):1333–47. doi: 10.1007/s11033-022-08063-z (PMC9889532; doi:10.1007/s11033-022-08063-z)
Supplement: Supplementary file 1 — Supplementary file1 (DOCX 1793 kb) [file 11033_2022_8063_MOESM1_ESM.docx]

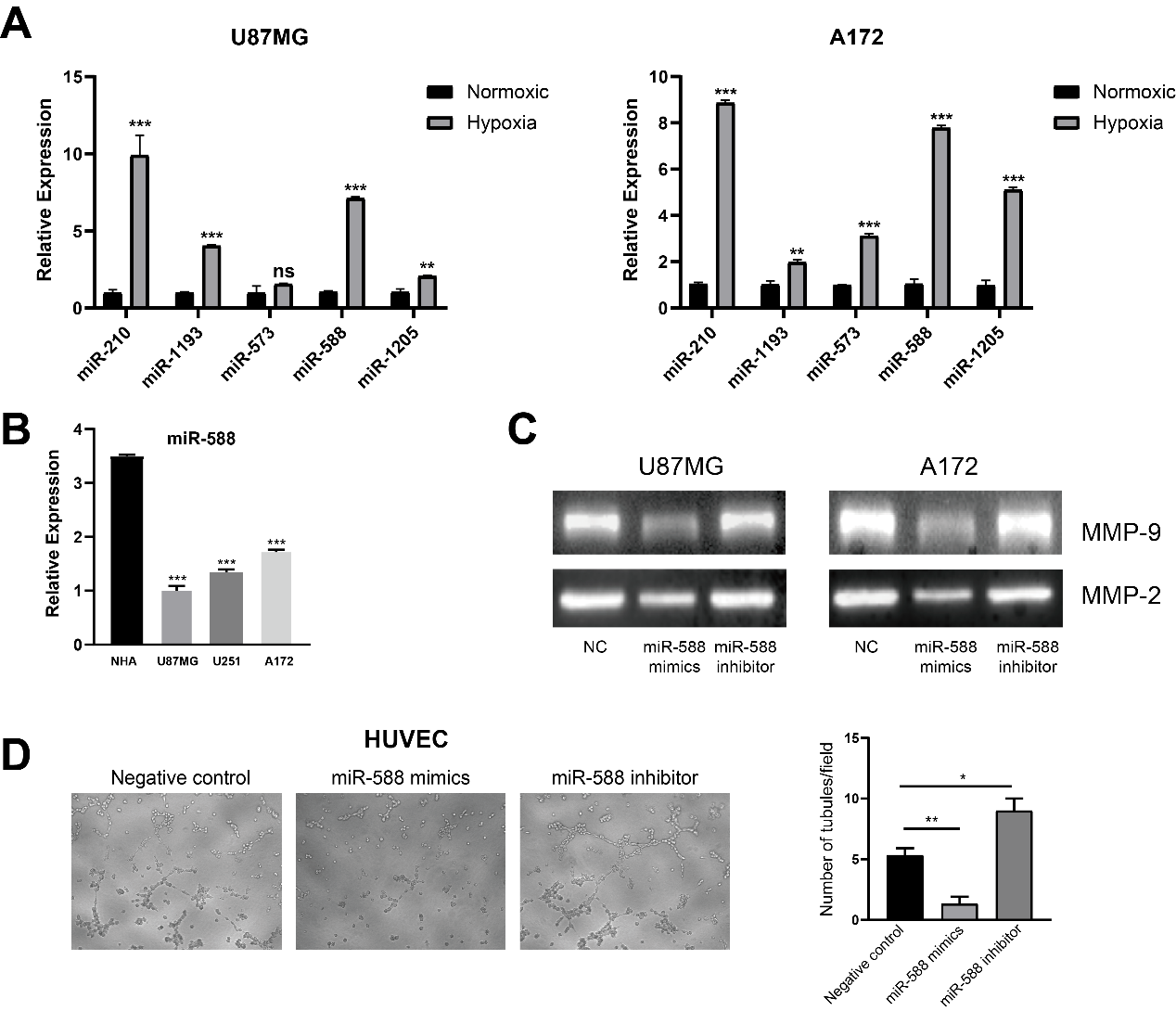


**Figure S1 A** The expression of candidate microRNAs detected by qRT-PCR. **B** The expression of miR-588 in normal human astrocytes (NHA) and glioma cell lines. **C** Detection of MMP-2 and MMP-9 enzymatic activities by gelatinase profiling assay. **D** The representative images and quantification of vasculogenic mimicry (VM)-forming ability of HUVEC.


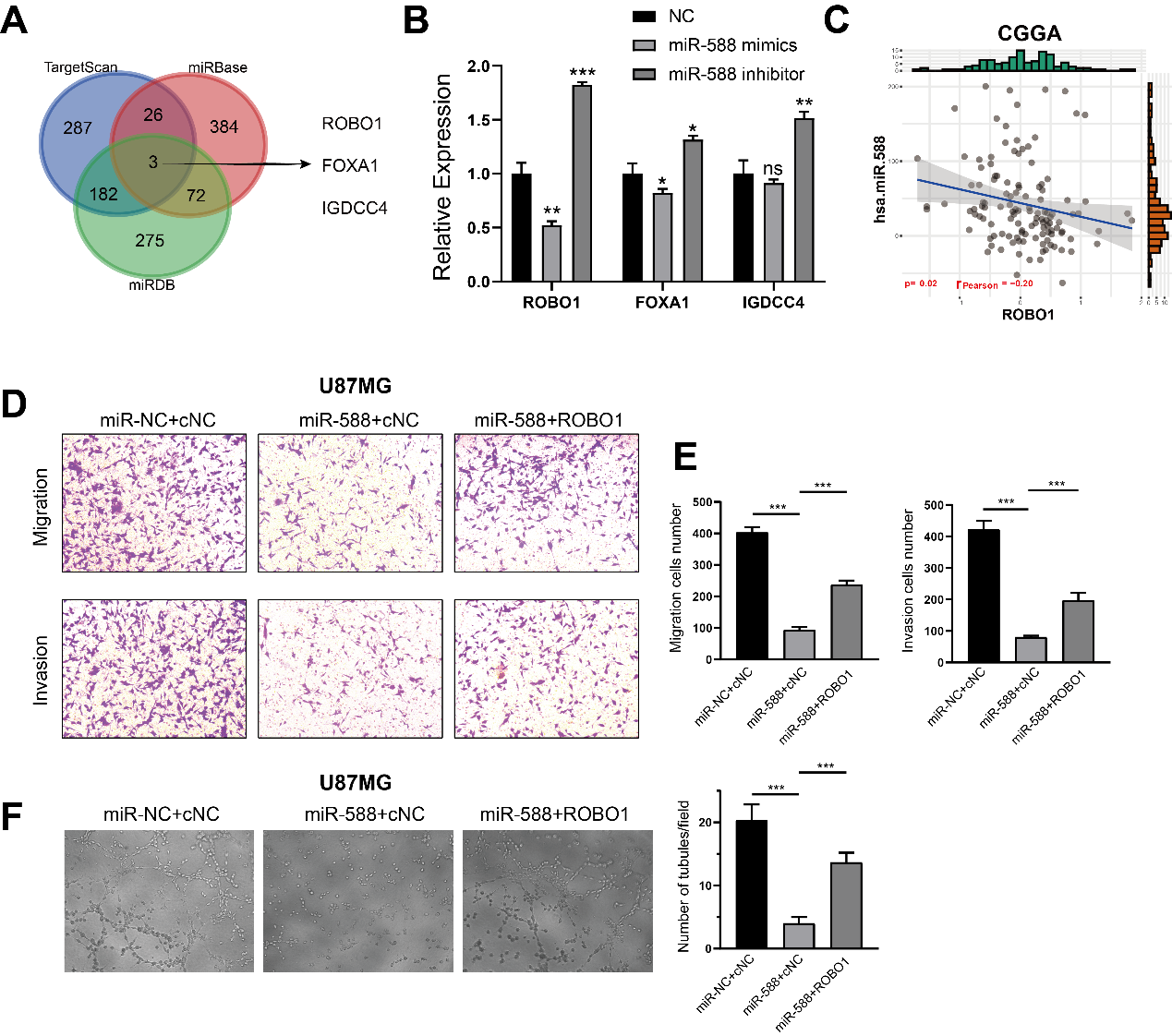


**Figure S2 A** The venn plot visualized the intersected microRNAs from there datasets. **B** The relative expression of ROBO1, FOXA1 and IGDCC4 detected by qRT-PCR. **C** Correlation analysis of miR-588 and ROBO1 in CGGA dataset. **D** The representative images of transwell experiment revealing the migration and invasion abilities of U87MG cells. **E** The quantification of transwell experiment. **F** The representative images and quantification of VM-forming experiments for U87MG cells.


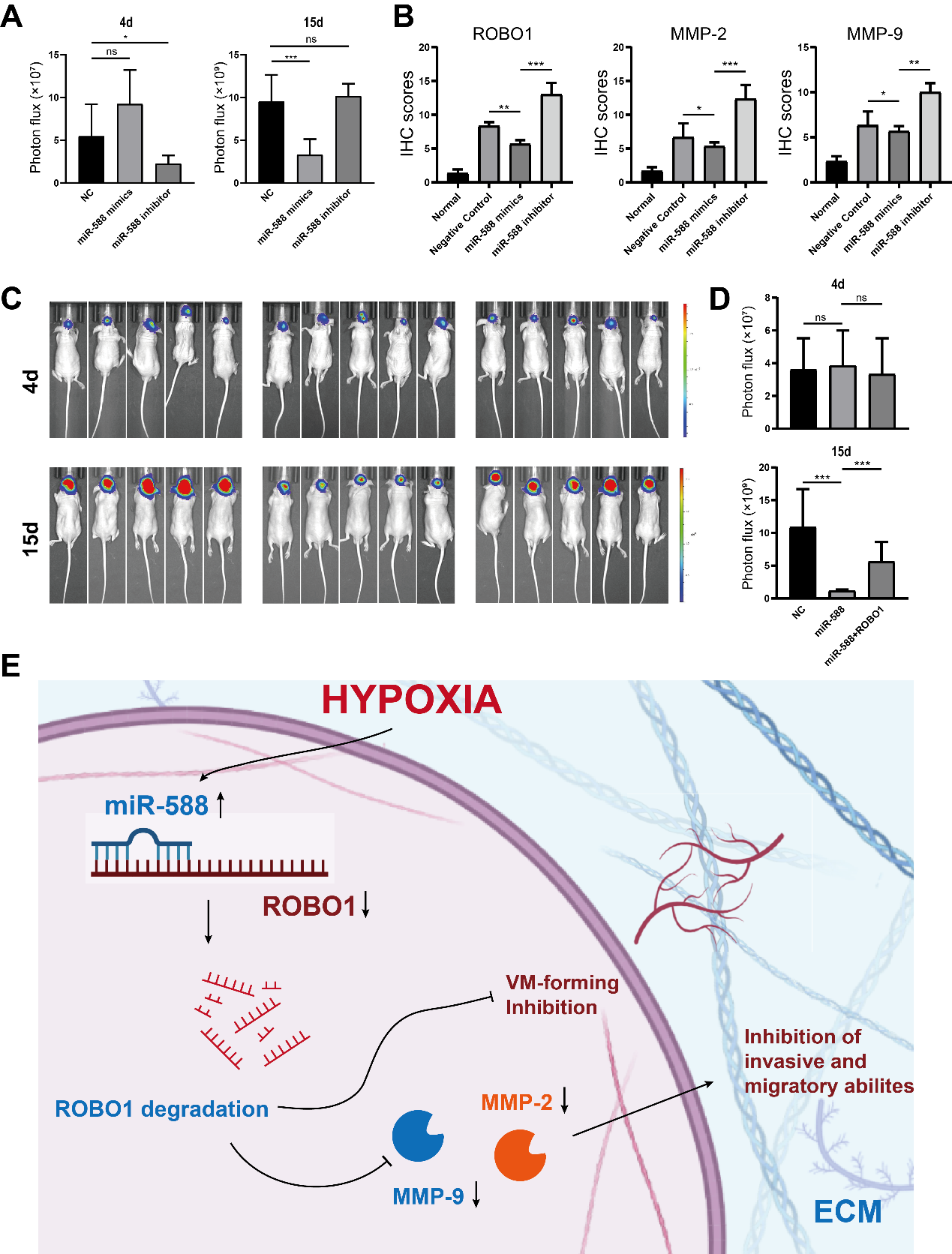


**Figure S3 A** The quantification of photon counts of U87MG xenografts on day 4 and day 15, respectively. **B** The quantification of IHC scores of sections from different xenografts. **C D** Bioluminescence imaging and quantification of tumor size of xenograft nude mice in different groups on day 4 and day 15, respectively. **E** The graphical model of this study.

**Supplementary Table**
